# Towards the automatic detection and correction of abnormal arterial pressure waveforms

**DOI:** 10.1007/s10877-024-01152-3

**Published:** 2024-04-04

**Authors:** Frederic Michard

**Affiliations:** MiCo, Vallamand, Switzerland

**Keywords:** Arterial pressure monitoring, Damping, Resonance, Systolic pressure, Cardiac output

## Abstract

Both over and underdamping of the arterial pressure waveform are frequent during continuous invasive radial pressure monitoring. They may influence systolic blood pressure measurements and the accuracy of cardiac output monitoring with pulse wave analysis techniques. It is therefore recommended to regularly perform fast flush tests to unmask abnormal damping. Smart algorithms have recently been developed for the automatic detection of abnormal damping. In case of overdamping, air bubbles, kinking, and partial obstruction of the arterial catheter should be suspected and eliminated. In the case of underdamping, resonance filters may be necessary to normalize the arterial pressure waveform and ensure accurate hemodynamic measurements.

## Introduction

Hirahata et al. [1] recently described a new and original method to detect underdamped arterial pressure waveforms and systolic pressure overshoot. Their study also confirmed the influence of tubing length on the magnitude of underdamping: the longer the tubing, the higher the likelihood of resonance phenomena. However, their model was experimental and did not address the additional influence the intra-arterial catheter may have in real-life conditions. Indeed, what they described as the “catheter” in their manuscript is the tubing, whose length ranged between 30 and 210 cm. Catheters used for intra-arterial pressure monitoring are typically 5–20 cm long and both their length and inner diameter influence arterial pressure waveform damping [2]. Nevertheless, the study by Hirahata et al. [1] is a useful reminder that abnormal arterial pressure waveforms are often overlooked at the bedside and may have a significant impact on the accuracy of hemodynamic variables [2, 3].

## The impact of abnormal damping on arterial pressure monitoring

Arterial catheters are mainly used for the continuous monitoring of arterial pressure in high-risk surgical and critically ill patients. In these patients, hypotensive events are frequent, and, if prolonged, may be responsible for tissue hypoperfusion and organ dysfunction. Therefore, the timely detection and correction of hypotensive events is a key element of effective hemodynamic management.

The overdamping of arterial pressure waveforms is responsible for an underestimation of systolic arterial pressure, whereas underdamping is responsible for an overestimation of systolic pressure [2, 3]. Underdamping is observed in up to one-third of critically ill patients [3]. Both overdamping and underdamping may lead to wrong therapeutic decisions when they are based on systolic pressure measurements. The mean arterial pressure (MAP) is known to be a better estimate of organ perfusion pressure and is the pressure used to calculate vascular resistances. The MAP also has the advantage of being relatively insensitive to damping phenomena [2, 3]. Therefore, one should rely on the MAP rather than on the systolic pressure to make therapeutic decisions.

## The impact of abnormal damping on other hemodynamic variables

Arterial pressure waveforms are increasingly used by pulse wave analysis techniques to compute stroke volume and cardiac output (CO). From the simultaneous measurements of MAP and CO, it is possible to assess vascular tone by calculating total vascular resistance (TVR = MAP/CO) or, when the central venous pressure (CVP) is also monitored, by calculating systemic vascular resistance (SVR = (MAP – CVP) / CO). Various algorithms have been developed and commercialized to compute CO from the arterial pressure waveform [4]. They are highly dependent on the morphological characteristics of pressure waveforms [4]. Until recently, the impact of abnormal waveforms on the accuracy of CO monitoring with pulse wave analysis techniques had not been studied. In critically ill patients with confirmed underdamped arterial pressure waveforms, Foti et al. [5] compared cardiac output measurements before and after the normalization of the pressure waveform with resonance filters. They reported a substantial overestimation (79 to 91%) of cardiac output with underdamped waveforms. The overestimation of CO may lead to the underdiagnosis of low-flow states and the undertreatment of hypovolemia and/or cardiac dysfunction. The overestimation of CO is also responsible for an underestimation of vascular tone (TVR = MAP/CO) and may be associated with the excessive use of vasopressors which have known side effects on microcirculation and end-organ perfusion [6].

The maximum rate of arterial pressure rise during systole (dP/dt_MAX_) has been proposed to assess left ventricular contractility [7] and to trigger the administration of inotropes [8, 9]. A recent study [10] showed that underdamping is responsible for a substantial (84% on average) increase in dP/dt_MAX_ values, which is > 5-fold higher than the systolic pressure overestimation. These findings may have significant clinical implications. For instance, a decrease in dP/dt_MAX_ may trigger the administration of inotropes whereas it may simply reflect an increase in damping (e.g. related to an air bubble in the tubing system). Unjustified administration of inotropes would expose patients to side effects such as cardiac arrhythmia and myocardial injury without any expected benefit. Conversely, an increase in dP/dt_MAX_ may be interpreted as an improvement in cardiac contractility whereas it may simply reflect a decrease in damping.

## How to detect abnormal damping?

The first method to detect or at least suspect abnormal damping is the visual inspection of the arterial pressure waveform. Overdamped waveforms are “flat” and the dicrotic notch is barely visible [2]. Nurses are very familiar with this issue which is often resolved by eliminating air bubbles or line kinking, or by flushing the catheter to eliminate partial obstruction. In contrast, underdamped waveforms are characterized by a sharp increase in systolic pressure (aka overshooting) that is not always identified by mere visual inspection [2, 3]. This systolic pressure overshoot is classically followed by a descending slope with multiple oscillations [2, 3].

In 1981, Gardner [11] proposed a method to detect and quantify abnormal damping. Flushing the crystalloid fluid that fills the tubing and transducer with high pressure (aka fast flush test) generates what is known as a system’s impulse response in signal processing. Right after the maneuver, oscillating waves fade exponentially with a pattern that depends on the damping coefficient. The natural frequency of the system is calculated by dividing the monitor speed by the wavelength of the oscillating waves. Then, the amplitude ratio of two consecutive resonant waves is calculated by dividing the amplitude of the smaller wave (second) by the amplitude of the higher one (first). Once the amplitude ratio is calculated, it is plotted against the natural frequency in a dedicated graph where three areas enable discrimination between adequate dynamic response, overdamping, and underdamping. However, this method has been criticized [12], and both the damping coefficient and natural frequency are not measured in routine practice.

The most popular method to easily detect abnormal damping at the bedside is the visual inspection of the pressure waveform at the end of a fast flush test [2]. When the arterial pressure waveform is normally damped, a couple of oscillations are visible at the end of the test. When the waveform is overdamped, no oscillations are observed, and when the waveform is underdamped, multiple oscillations are visible [2]. This later pattern, characteristic of underdamped waveforms, is known as the “ringing effect”.

## Towards the automatic detection and correction of abnormal arterial pressure waveforms

**Fig. 1:**
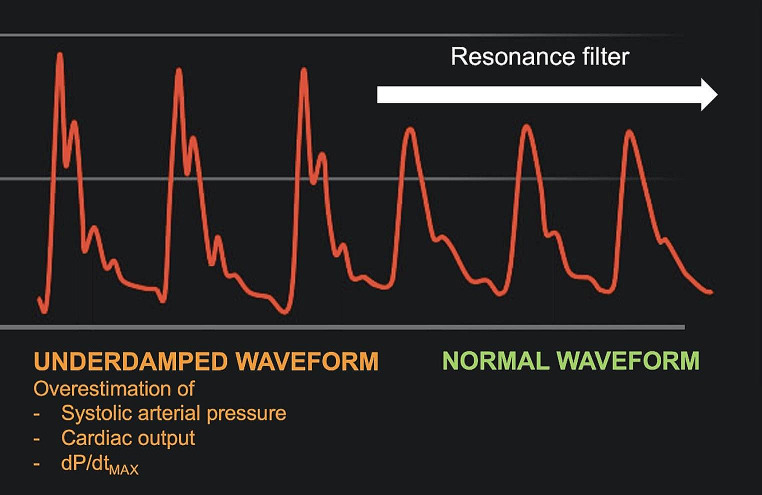
Example of underdamped arterial pressure waveform and the automatic filtering of resonance phenomena with an electronic filter

Algorithms have recently been developed to automatically detect and/or correct abnormal arterial pressure waveforms. Rinehart et al. [13] tested a machine learning algorithm trained to detect overdamping. In 38 surgical patients monitored with a radial arterial catheter, they induced overdamping by adding an air bubble into the arterial blood pressure fluid line. Their algorithm was able to detect overdamping with a sensitivity of 98% and a specificity of 92%. Another algorithm has been developed to automatically detect and correct underdamping (Fig. 1). In 70 critically ill patients with underdamped arterial pressure waveforms (confirmed by the Gardner method), this new algorithm (or electronic filter) was able to provide systolic arterial pressure and CO values comparable to those obtained with a mechanical resonance filter [5]. This electronic filter is commercially available and has already been used in several clinical studies reporting a good agreement between CO measurements from pulse wave analysis and thermodilution [14, 15].

## Conclusion

Both over and underdamping are frequent during continuous invasive arterial pressure monitoring. They may influence arterial pressure measurements (mainly systolic pressure) and the accuracy of CO monitoring with pulse wave analysis techniques. It is therefore recommended to regularly perform fast flush tests to unmask abnormal damping. Smart algorithms have recently been developed for the automatic detection of abnormal damping. In case of overdamping, kinking, air bubbles, and partial obstruction of the arterial catheter should be suspected and eliminated. In the case of underdamping, the use of resonance filters may be necessary.

## Data Availability

No datasets were generated or analysed during the current study.

## Abbreviations

CO: cardiac output
CVP: central venous pressure
dP/dt_MAX_: maximum rise of the arterial pressure during systole
MAP: mean arterial pressure
SVR: systemic vascular resistance
TVR: total vascular resistance
